# Marrow to Bowel or Bowel to Marrow: Intestinal Myeloid Sarcoma Presenting With Acute Obstruction

**DOI:** 10.7759/cureus.113092

**Published:** 2026-07-21

**Authors:** Vipul Bhatt, Ambika Kaushal, Prerna Arora, Shramana Mandal, Matiz Hossain, Chandra Bhushan Singh

**Affiliations:** 1 Pathology, Maulana Azad Medical College, Delhi, IND; 2 Surgery, Maulana Azad Medical College, Delhi, IND

**Keywords:** acute myeloid leukemia, extramedullary leukemia, gastrointestinal tract, intestinal obstruction, myeloid sarcoma

## Abstract

Myeloid sarcoma (MS) is an extramedullary tumor composed of immature myeloid precursor cells and represents a tissue manifestation of acute myeloid leukemia (AML). It may occur concurrently with AML, precede marrow disease, or present at relapse. Gastrointestinal (GI) involvement is uncommon and frequently poses a diagnostic challenge due to morphological overlap with lymphoid and other GI malignancies. We report the case of a 38-year-old man presenting with acute intestinal obstruction secondary to a duodenal-jejunal mass. Histopathological examination demonstrated diffuse submucosal infiltration by monomorphic abnormal cells, initially raising a differential diagnosis of lymphoma or poorly differentiated malignancy, intestinal tuberculosis, or gastrointestinal stromal tumor. Immunohistochemistry revealed strong myeloperoxidase positivity, with negativity for epithelial, lymphoid, and neuroendocrine markers, supporting myeloid lineage. Peripheral blood examination showed leukocytosis with circulating blasts and monocytic precursors. Multiparametric flow cytometry was suggestive of AML with monocytic differentiation, establishing the diagnosis of intestinal MS with concurrent AML. The present case highlights the rarity of MS presenting as an acute abdomen and surgical emergency with diagnostic challenges to the surgeon. Accurate diagnosis relies on keeping a high index of clinical suspicion, along with a multidisciplinary approach. Early identification is critical, as MS requires prompt AML-directed systemic therapy rather than localized surgical treatment.

## Introduction

Myeloid sarcoma (MS) is a rare extramedullary tumor composed of immature myeloid precursor cells. According to the 2022 World Health Organization classification, MS is recognized as the tissue counterpart of acute myeloid leukemia (AML), myelodysplastic neoplasms, or myeloproliferative neoplasms and often reflects aggressive clonal evolution with leukemic dissemination [1]. Genetic mutations in myeloid progenitors lead to the clonal expansion of myeloid blasts and escape into the blood with local infiltration and homing in various solid organs via adhesion molecules and chemokines, thereby leading to the formation of MS. The reported incidence of MS varies widely, with clinical studies documenting a prevalence ranging from 7% to 30% in AML cohorts, while population-based Surveillance, Epidemiology and End Results analyses emphasize its rarity but prognostic significance [2,3]. MS commonly involves the skin, lymph nodes, soft tissue, and, less frequently, visceral sites such as the central nervous system, kidney, lung, pancreas, female genital tract, and gastrointestinal tract (GIT) [2,4,5].

GIT MS is distinctly uncommon and is often misdiagnosed as surgical diseases due to the common presentation as a mass lesion, GIT bleeding, or acute intestinal obstruction, while overt presentation as leukemia is rare. GIT MS accounts for <15% of reported cases, with duodenal involvement being infrequent [2]. Diagnostic difficulty in GIT MS arises due to nonspecific imaging findings and morphological overlap with lymphoma, poorly differentiated carcinoma, neuroendocrine tumors, and gastrointestinal stromal tumor (GIST) [4,6]. Isolated or aleukemic MS is particularly prone to misdiagnosis as they present only as extramedullary masses initially and is reported to precede overt AML in a significant proportion of patients [3].

Indian literature on MS remains limited, with most reports describing orbital, lymph nodal, and cutaneous involvement. In contrast, GIT MS is rarely documented, possibly reflecting lower biopsy rates and constraints in immunophenotyping with significant overlap with other differentials. Thus, the diagnosis of GIT MS may remain elusive and misdiagnosed; hence, a high clinical index of suspicion should be maintained for the rare diagnosis of MS even with a common GIT presentation. Herein, we report a rare case of duodenal and jejunal MS in a young man, along with a review of relevant literature.

## Case presentation

**Figure 1:**
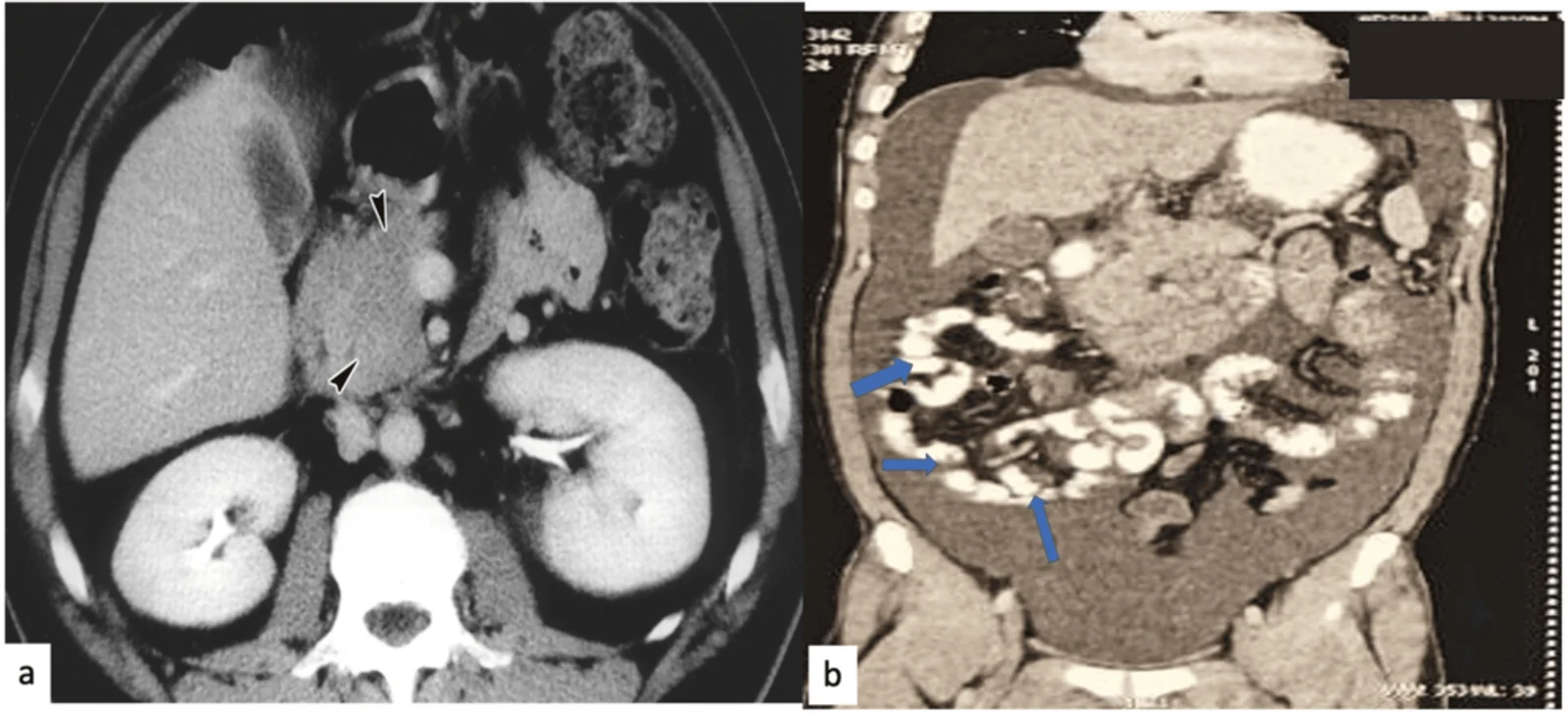
Contrast-enhanced computed tomography (CECT) of the abdomen. (a) CECT of the abdomen revealing a retroperitoneal mass lesion (black arrowhead). (b) CECT of the abdomen showing multiple mural wall thickenings in the small intestine leading to stricture and luminal narrowing (blue arrows).

A 38-year-old man presented with acute-onset abdominal pain radiating to the back, associated with vomiting and obstipation for five to six days. There was no history of fever, weight loss, jaundice, or gastrointestinal (GI) bleeding. On examination, the patient’s vitals were stable, with a pulse rate of 72 beats/minute and a blood pressure of 110/70 mm Hg. Pallor and abdominal distension with ascites were also noted. No peripheral lymphadenopathy was identified. An ultrasound of the abdomen revealed mesenteric and retroperitoneal lymphadenopathy (the largest node measuring 14 mm with the loss of the fatty hilum), moderate ascites, diffuse bowel wall thickening, and omento-mesenteric haziness. Contrast-enhanced computed tomography of the abdomen revealed an ill-defined homogeneous retroperitoneal soft-tissue mass with circumferential mural thickening involving the third and fourth parts of the duodenum and proximal jejunum, resulting in marked luminal narrowing and gastric outlet obstruction (Figures 1a, 1b).

Associated findings included acute interstitial pancreatitis and bilateral pleural effusions. Differential diagnoses included abdominal tuberculosis, intussusception, lymphoma, adenocarcinoma, and GIST. Laboratory investigations showed normal metabolic and liver function parameters, with raised serum creatinine, serum lactate dehydrogenase, and C-reactive protein. Table 1 depicts the biochemical parameters.

**Table 1:** Hematological and biochemical parameters at admission. AST = aspartate transaminase; ALT = alanine transaminase; ALP = alkaline phosphatase; EBV = Epstein-Barr virus; HIV = human immunodeficiency virus

Parameter	Results	Reference range
Hemoglobin	10.3 g/dL	13–17 g/dL
Total leucocyte count	28,750/µL	4,000–11,000/mm^3^
Platelet count	90,000/µL	150,000–400,000/µL
Blast equivalents (monocytoid)	36%	Nil
Serum bilirubin	0.4	0.30–1.20 mg/dL
Total protein	6.2	6.0–8.0 g/dL
AST	15	10–40 U/L
ALT	20	10–40 U/L
ALP	120	30–115 U/L
Sodium	134	135–145 mEq/L
Potassium	3.0	3.5–5.3
Serum creatinine	3.4 mg/dL	0.7–1.3 mg/dL
Serum lactate dehydrogenase	500 U/L	140–280 U/L
C-reactive protein	24 mg/L	<5 mg/L
Viral serology	Non-reactive	Non-reactive
HIV	Non-reactive	Non-reactive
Hepatitis B and C	Non-reactive	Non-reactive
Cytomegalovirus	Non-reactive	Non-reactive

**Figure 2:**
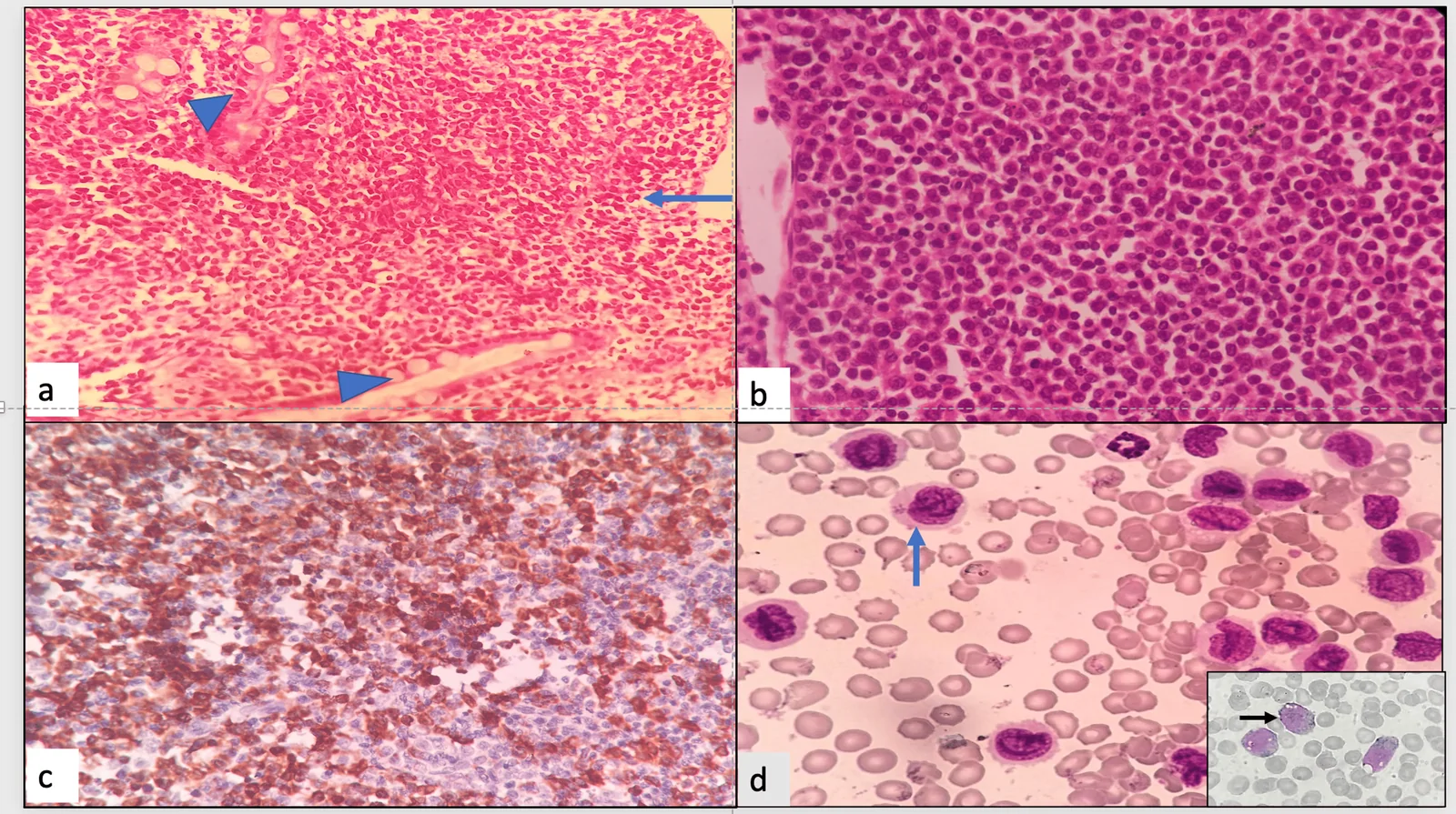
Histopathology and peripheral smear. (a) Duodenal-jejunal biopsy showing diffuse submucosal infiltration by monomorphic atypical cells arranged in sheets (blue arrow). The cells exhibited round-to-oval nuclei, a high nuclear-to-cytoplasmic ratio, open chromatin and scant cytoplasm, with the presence of normal mucosal glands (blue arrowhead ) (hematoxylin and eosin: ×200). (b) Retroperitoneal lymph node with the effacement of architecture by similar atypical cells (hematoxylin and eosin: ×200). (c) Tumor cells showing diffuse positivity for leukocyte common antigen (immunohistochemistry: ×200). (d) Peripheral smear showing blasts and monocytoid blast equivalents with irregular nuclear contours, pale basophilic cytoplasm, and prominent nucleoli (blue arrow) (Giemsa stain, ×1,000; oil immersion). Inset: cytochemistry showing myeloperoxidase positivity, confirming myeloid lineage blasts (black arrow).

Given the clinical presentation and imaging findings suggestive of intestinal obstruction, an exploratory laparotomy was undertaken. Intraoperatively, a circumferentially thickened and indurated segment of proximal jejunum was noted approximately 30-40 cm distal to the duodenal-jejunal flexure, with proximal small bowel dilatation. The adjacent mesentery was bulky, harboring multiple enlarged firm lymph nodes. The lesion extended proximally toward the distal duodenum (D3/D4). In addition, strictures were found at multiple levels in the small intestine. No intraoperative evidence of intussusception or a discrete exophytic mass was observed. The patient was initially planned for gastrojejunostomy in view of acute gastric outlet obstruction; however, it could not be performed because of multiple small bowel strictures. Further, small bowel resection also could not be performed due to the involvement of the entire small bowel. Hence, a palliative gastrostomy was undertaken, and representative biopsies from the duodenum, jejunal wall, and mesenteric lymph nodes were obtained. The histopathological evaluation of duodenal and jejunal biopsies showed diffuse submucosal infiltration by monomorphic atypical cells arranged in sheets (Figure 2a).

**Figure 3:**
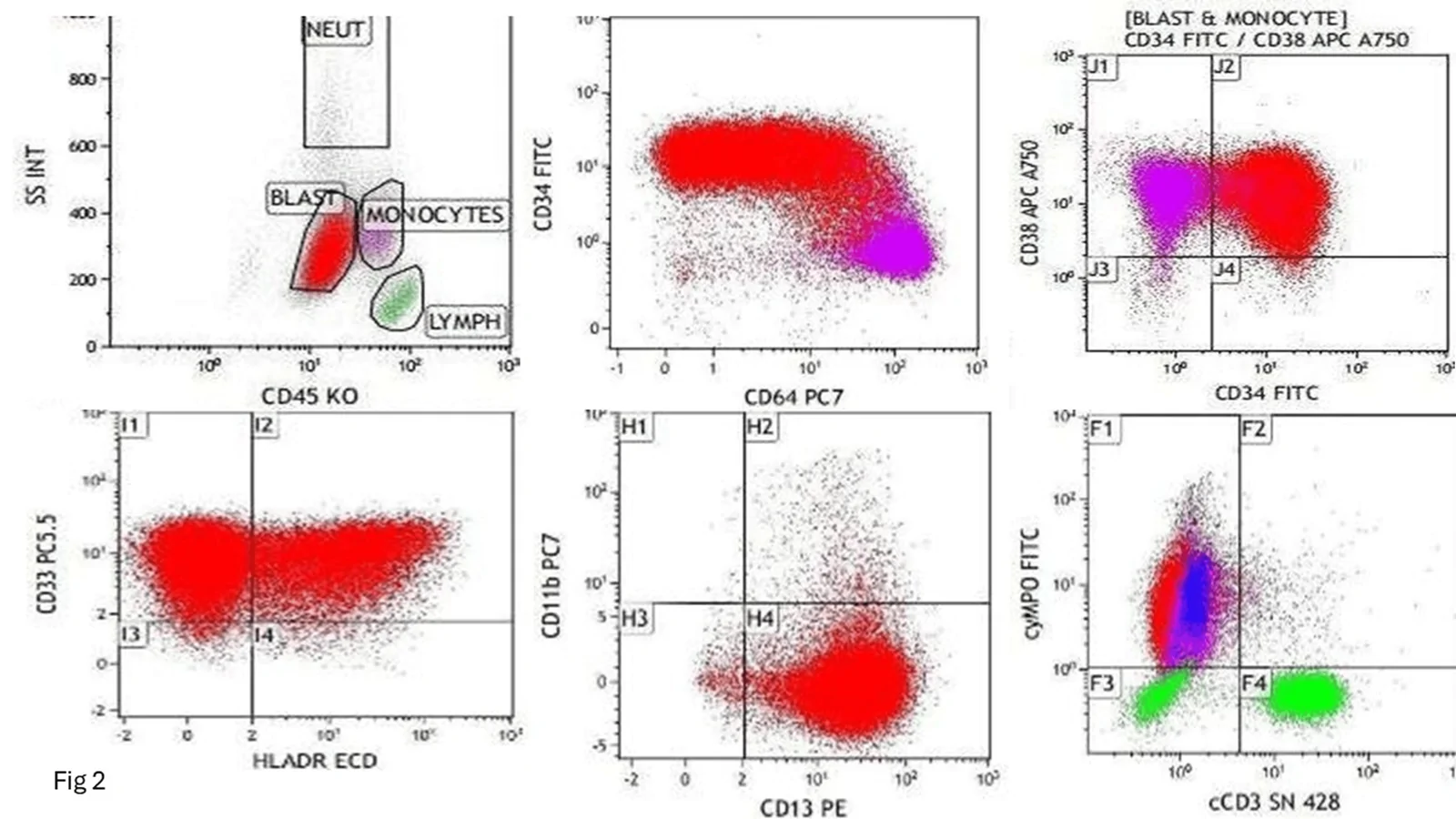
Flow cytometry. Flow cytometry showing (~73%) circulating blasts (red color), expressing myeloid markers CD34, CD64, CD38, CD33, HLA-DR, CD13, and myeloperoxidase and the absence of B- and T-lineage markers.

The differential diagnosis included lymphoma, GIST, poorly differentiated carcinoma, mesenchymal tumor, and metastasis. A retroperitoneal lymph node biopsy also revealed the effacement of nodal architecture by similar atypical cells (Figure 2b). Immunohistochemistry (IHC) panel demonstrated diffuse positivity for leukocyte common antigen (LCA) (Figure 2c), while epithelial markers (epithelial membrane antigen (EMA) and cytokeratin (CK)), neuroendocrine markers (chromogranin and synaptophysin), melanocytic marker (HMB45), vimentin, and c-Kit were negative. B- and T-cell markers (CD19 and CD3) were also negative, raising suspicion of a non-lymphomatous hematological malignancy. Subsequently, a complete hematological evaluation was advised, which revealed anemia and thrombocytopenia with the presence of 36% abnormal monocytoid precursors considered to be blast equivalents in the peripheral smear (Table 1, Figure 2d). Cytochemical stains demonstrated positivity for myeloperoxidase (MPO) and neuron-specific enolase, with weak periodic acid-Schiff positivity (Figure 2d, inset). On multiparametric flow cytometry, blasts expressed MPO, CD13, and CD33, along with monocytic markers CD14, CD64, and HLA-DR. CD34 and CD117 showed heterogeneous expression. B-lineage markers (CD10, CD19, CD20, and CD79a) and T-lineage markers (surface and cytoplasmic CD3, CD5, CD7, and CD8) were negative. These findings supported the diagnosis of AML with monocytic differentiation (AML-M5) (Figure 3).

A second IHC panel on intestinal biopsy showed positivity for MPO, CD13, CD33, and CD56, with a high proliferative index (Ki-67: 80%-90%). The correlation of hematological, histopathological, immunohistochemical, cytochemical, and flow cytometric findings established the diagnosis of intestinal MS with concurrent AML.

Postoperatively, the patient’s clinical condition deteriorated rapidly, with progressive leukocytosis and sepsis, and he succumbed within one week of diagnosis due to leakage from the jejunostomy site and septic shock.

## Discussion

MS is a rare extramedullary manifestation of systemic AML, either at initial presentation or during relapse; however, a clinically meaningful subset presents as isolated or aleukemic MS, without evident marrow involvement. Several reviews have highlighted that isolated MS may precede the development of overt AML and carries a substantial risk of leukemic transformation and misdiagnosis [6-8]. In contrast, MS occurring in patients with established AML reflects disseminated disease at onset, although outcomes in both groups are influenced more strongly by underlying cytogenetic and molecular characteristics than by the anatomic site of involvement [7-10]. In the present case, the patient was diagnosed with MS with concurrent AML and presented with an acute surgical abdomen. GI MS accounts for approximately 10% of MS cases, with the small intestine being an uncommon site [5-7]. Further studies have confirmed that within the intestinal tract, the ileum is the most commonly affected part, accounting for approximately 65% of cases, while the duodenum and jejunum are rarely involved sites of MS [10]. However, the absolute incidence of GI MS is low, and its proportion among small intestinal tumors remains unknown. The present case had a mass lesion diagnosed as MS confined to the duodenum and jejunum, along with retroperitoneal lymphadenopathy.

MS of the small intestine is a rare but clinically significant entity and a great mimicker that can pose significant diagnostic and therapeutic challenges, especially to the treating surgeon and pathologists. Among GIT sites, gastric and duodenal MS are uncommon but well documented [2,4,6]. The present case illustrates several of these diagnostic challenges with the initial radiological and clinical impression, which simulated a lymphoma-like or carcinoma-like mass due to overlapping features [2,4]. The immunophenotypic profile in our case, with strong MPO expression and the absence of lymphoid, epithelial, and neuroendocrine markers, is concordant with the diagnostic framework proposed by Avni and Koren-Michowitz, who emphasized MPO as a key indicator of myeloid differentiation even when histology is ambiguous [5].

Multiple studies have also highlighted the need for a broad IHC panel, incorporating markers such as MPO, lysozyme, CD117, CD34, CD43, and CD68, particularly in GI lesions with nonspecific morphology, to avoid misinterpretation as lymphoma or poorly differentiated carcinoma [7,8]. Cytogenetics and molecular analysis workup, especially in extramedullary MS, also helps in establishing the diagnosis of an underlying clonal etiology and distinguishing it from other common morphological mimickers. Although our study lacked molecular analysis, the presence of circulating blasts, along with flow cytometric confirmation, supported the diagnosis of AML with monocytic differentiation, a subtype commonly associated with extramedullary MS. The evaluation of molecular abnormalities in MS also influences the prognosis and risk stratification, thereby aiding in therapeutic decision-making, not limited to only surgical intervention; hence, genetic workup is an essential part of the workup in MS, especially at rare and uncommon locations such as the GIT. Studies have also shown a high degree of cytogenetic concordance between MS lesions and corresponding marrow clones, reinforcing the concept that MS represents a tissue manifestation of systemic AML biology [10]. Zhao et al. (n = 118) further demonstrated associations with inv(16), t(8;21), KMT2A rearrangements, and *NPM1* mutations, abnormalities frequently linked to extramedullary disease (EMD) [9].

In line with current consensus recommendations, several authors advocate AML-type systemic chemotherapy as the cornerstone of treatment in both isolated and AML-associated MS, whereas local therapies such as surgery or radiotherapy alone do not prevent systemic progression and may delay definitive management [1,6-9]. Batah et al. reported that while patients with MS-associated AML may have lower complete remission rates, overall survival and event-free survival can approximate those of AML without EMD when treated with systemic chemotherapy, underscoring the importance of early recognition and timely therapy [11]. Extramedullary relapses are common in patients with MS; hence, patients need to be regularly followed up to confirm complete remission. Hence, a correct diagnosis of an unusual tumor type at an unusual site can change the management from surgical intervention to an aggressive medical approach. Table 2 illustrates various case series of reported MS involving the GIT.

**Table 2:** Distribution of cases of GIT myeloid sarcoma in the previous case series. GIT = gastrointestinal tract; MS = myeloid sarcoma

Author (year)	Total number of MS cases	Most common site/s	Presenting complaints of GIT MS	Treatment
Zhao et al., 2022 [9]	118	GIT (4/118)	Mass lesion	Surgery + chemotherapy
Abedi et al., 2023 [12]	5	Scalp, nose, cecum, genital area, and mediastinal mass	Abdominal mass and obstruction	Chemotherapy
Thingujam et al., 2024 [13]	3	Small intestine	Abdominal mass and subacute intestinal obstruction	Surgery + chemotherapy
Rahman et al., 2024 [14]	6	Gingiva, stomach, lymph node, finger, eye, and lung	Mass lesion	Surgery + chemotherapy
Padmanabhan et al., 2025 [15]	7241	GIT (10%)	Variable	Surgery + chemotherapy
Present case	1	Small intestine	Gastric outlet and acute intestinal obstruction	Palliative surgery; no further treatment as the patient expired

## Conclusions

GI MS is a rare and diagnostically challenging presentation, and delay in diagnosis can worsen the outcome. This case highlights the importance of maintaining a high index of suspicion for MS in atypical abdominal masses. The clinical and pathological findings in our patient parallel observations from global literature, though further studies in larger cohorts are needed to better elucidate the pathophysiology of GIT MS. Early initiation of AML-directed systemic chemotherapy in MS remains essential. Given the scarcity of GIT MS reports in Indian cohorts, this case adds valuable regional experience and underscores the importance of early and multidisciplinary collaboration among surgeons, radiologists, and pathologists, combined with comprehensive immunophenotyping, for timely diagnosis and initiation of systemic therapy, thereby optimizing patient outcomes, as not every intestinal obstruction has a surgical cause.
